# Effects of aeration of softwood pretreatment liquid on inhibitors and fermentability using *Saccharomyces cerevisiae* yeast

**DOI:** 10.1186/s13068-025-02708-4

**Published:** 2025-10-08

**Authors:** Chaojun Tang, Carlos Martín, Leif J. Jönsson

**Affiliations:** 1 Department of Chemistry, Umeå University, 901 87 Umeå, Sweden; 2 Department of Biotechnology, Inland Norway University of Applied Sciences, N-2317 Hamar, Norway

**Keywords:** Lignocellulose, Lytic polysaccharide monooxygenase, Aeration, Inhibitors, Formaldehyde, Methanol, Detoxification, Laccase, Sulfite

## Abstract

**Background:**

Aeration plays a critical role in the bioconversion of pretreated lignocellulose by enhancing lytic-polysaccharide-monooxygenase(LPMO)-supported enzymatic saccharification. However, its broader impact, particularly on fermentation inhibitors, remains insufficiently understood. The hypothesis that aeration not only promotes LPMO activity, which has been shown clearly in previous studies, but also affects fermentation inhibitors was investigated in experiments with softwood pretreatment liquids. The effects of aeration were explored through chemical analysis of fermentation inhibitors and through subsequent fermentations with the xylose-utilizing *Saccharomyces cerevisiae* yeast CelluX™4 to test the fermentability. Controls in which N_2_ rather than air was supplied to the pretreatment liquids were used to distinguish between evaporation effects and effects caused by oxidation due to O_2_ in air. In separate experiments, two redox-dependent detoxification methods, treatments with sulfite and laccase, were further investigated.

**Results:**

While aeration had no negative effects on the subsequent fermentation of a sugar control, it compromised the fermentability of the pretreatment liquids. Compared to the N_2_ control, subsequent fermentation of aerated samples showed reduced consumption of fermentable sugar (glucose, mannose, xylose) at 0.61 compared to 0.76 g L^−1^ h^−1^, and lower ethanol productivity (0.23 vs. 0.30 g L^−1^ h^−1^). Apart from more commonly studied pretreatment by-products (such as aliphatic carboxylic acids, furan aldehydes, and phenolics), methanol (~ 1 g L^−1^) was detected in both pretreatment liquids. The methanol concentration decreased during gas addition, which was attributed to evaporation. Compared to the initial pretreatment liquid, aerated reaction mixtures exhibited slightly elevated levels of formaldehyde, but lower levels of furfural and vanillin. Sulfite detoxification was successful under both aeration and N_2_ conditions. Treatment with laccase was found to have variable effects on the fermentability depending on the conditions applied.

**Conclusions:**

The results underscore the dual role of aeration in softwood bioconversion, positive for promoting LPMO activity but potentially negative with respect to subsequent fermentability, and highlight the need to carefully tailor aeration strategies for the design of efficient biochemical processing of lignocellulosic feedstocks. Treatment with reducing agents, such as sulfite, emerges as a possibility to alleviate negative side-effects on the fermentability when aeration is used to promote LPMO activity.

## Background

Replacing petroleum and other fossil resources with sustainable and renewable lignocellulosic feedstocks has gained significant attention [1]. Renewable energy plays a vital role in the global approach to the mitigation of environmental issues caused by extensive use of fossil resources, and is valuable from an energy security point of view. Bioenergy, including forest bioenergy, can play a significant role in climate change mitigation by displacing fossil fuels and supporting energy system transition [2]. Bioethanol from forest-industrial residues, such as sawdust, is of interest for various applications, which includes serving as a biofuel intermediate in the production of sustainable aviation fuel [3].

Biochemical conversion of biomass typically involves acid pretreatment prior to enzymatic saccharification, fermentation of sugars, and valorization of the lignin that is left after degradation and solubilization of hemicelluloses and cellulose [4–7]. Pretreatment under acidic conditions may involve steam explosion for improved disintegration of fibers. Although other constituents are also affected, acid pretreatment primarily targets hemicelluloses, which greatly facilitates the accessibility of enzymes to cellulose. Sulfur-dioxide-assisted steam explosion is a state-of-the-art pretreatment method that can effectively be applied to softwood, rendering highly reactive cellulose for saccharification and releasing hemicellulosic sugars, primarily hexoses, that can be fermented by ethanologenic microorganisms [8]. Studies of pretreatment conditions for softwood and how they affect enzymatic digestibility and fermentability have been reported by Wang et al. [8].

Pretreatment also results in side reactions that create unwanted by-products that are inhibitory to downstream biochemical processes. By-products that can inhibit microbial fermentation processes include aromatic substances (such as phenolics), furan aldehydes [such as furfural and HMF (5-hydroxymethylfurfural)], aliphatic acids (such as acetic acid), small aliphatic aldehydes (such as formaldehyde and acetaldehyde), and benzoquinones [9, 10]. The levels of the inhibitors depend on raw materials, pretreatment process, pretreatment conditions, and solids loadings. There are different approaches to deal with inhibitors, including conditioning, i.e., detoxification of slurries and hydrolysates using various methods [11]. These include, among many others, the use of the phenol oxidase laccase [12–14] and the use of reducing agents, including sulfite [10, 15].

Regarding the enzymatic saccharification of cellulose, hydrolytic enzymes, such as endoglucanase, cellobiohydrolase, and β-glucosidase, are commonly used [16]. In addition to the hydrolytic enzymes, the oxidoreductase LPMO (lytic polysaccharide monooxygenase) has emerged as a new tool in enzymatic saccharification of cellulosic substrates [17, 18]. Unlike classic glycosyl hydrolases, which possess obvious substrate-binding grooves, LPMO utilizes a relatively flat surface for substrate interaction. The active site features a copper ion coordinated with two His in a histidine brace, one Tyr, and two water molecules [19]. This structural arrangement enables the oxidative cleavage of polysaccharides, such as cellulose, by LPMOs. In the presence of molecular oxygen and an electron donor, LPMO serves as a complement to the hydrolytic enzymes due to its ability to catalyze cleavage of glycosidic bonds in cellulose, thereby creating more targets for glycosyl hydrolases. Various substances can serve as electron donors, but from a process-oriented viewpoint it is especially relevant that lignin and lignin-degradation products can have that function [20, 21].

Although there are alternatives, *Saccharomyces cerevisiae*, i.e., baker's yeast, is commonly used for fermentation of sugars [22]. *S. cerevisiae* is a preferred choice due to its capacity to provide a high product yield and due to its well-established role in industrial fermentation for bioethanol production. Oxygen availability would normally influence glycolytic metabolism, as the presence of oxygen typically promotes aerobic respiration, in which pyruvate is fed into the TCA (tricarboxylic acid) cycle resulting in oxidative phosphorylation with high ATP yield and efficient energy conversion. Anaerobic conditions would instead result in fermentation, and, depending on the organism, result in products such as ethanol and lactic acid, with low yield of ATP. However, when sugar concentrations are high enough, *S. cerevisiae* can ferment under both aerobic and anaerobic conditions, known as the Crabtree effect [23]. Thus, *S. cerevisiae* has a preference for alcoholic fermentation even when oxygen is present and respiration would be the normal mode of energy metabolism [23]. Although *S. cerevisiae* does not ferment xylose, which is typically an abundant monosaccharide in hydrolyzed biomass, there are strains that have been engineered for improved utilization of xylose [22].

In an industrial scenario, saccharification and fermentation can be carried out using different process configurations [5, 6]. A standard approach is to use separate hydrolysis and fermentation (SHF), in which enzymatic saccharification and microbial fermentation are performed sequentially. This allows the use of optimal conditions in both steps. Simultaneous saccharification and fermentation (SSF) is an alternative, in which enzymatic saccharification and fermentation are performed concomitantly. SSF offers advantages such as reduced end-product inhibition of cellulolytic enzymes by sugars, but also suffers disadvantages such as process conditions being a compromise between the optima of the saccharification and the fermentation reactions. The choice of processing strategy is affected by the potential use of LPMO in enzyme preparations [24]. One approach is to use hybrid hydrolysis and fermentation (HHF) [25, 26], in which there is an enzymatic pre-hydrolysis step before the yeast is added. During the pre-hydrolysis, it is possible to use aeration to improve saccharification by supplying oxygen to drive the LPMO-catalyzed reaction and use high temperatures that yeast would not be able to withstand but which are well suited for thermotolerant enzymes. However, a recent study of HHF with pretreated softwood showed surprisingly poor fermentability after the pre-hydrolysis step [26]. Potential consequences of aerated pre-hydrolysis, apart from improved saccharification of cellulose by promoting LPMO [27], were therefore further investigated in the present study.

Apart from promoting LPMO, aeration could potentially affect pretreatment by-products, including inhibitors, by oxidation, but also by evaporation if a stream of gas is applied during saccharification. Also, some detoxification methods are based on redox reactions, including treatment with reducing agents [10], such as sulfite, and enzymatic treatment with the phenol oxidase laccase [12–14].

While effects of aeration on LPMO activity and biomass convertibility have been explored in several previous studies [21, 26, 27], this work was instead focused on potential effects of aeration on pretreatment by-products including inhibitors. This was studied using pretreatment liquids obtained from steam explosion of sawdust of softwood (Norway spruce) and exploring how inhibitor levels were affected by aeration and how the subsequent fermentability after the aeration step was affected. The fermentability was assessed using a state-of-the-art engineered xylose-utilizing *S. cerevisiae* yeast, CelluX™4. To account for changes caused by gas addition in general, and oxygenation in particular, parallel treatments were carried out using air and N_2_. Two redox-based detoxification methods were also studied: treatment with sodium sulfite and treatment with laccase. With respect to treatment with reducing agents, such as sulfite, there is a risk that aeration depletes the reducing agent so that it becomes less effective. Also, if aeration causes accumulation of toxic inhibitors by oxidation, there is a possibility that treatment with reducing agents can alleviate that. Laccase treatment was studied because its catalysis is driven by molecular oxygen. Thus, the risk that aeration causes accumulation of certain inhibitors by oxidation could possibly occur also during laccase treatment. For example, this could potentially happen if toxic benzoquinone were formed through oxidation of hydroquinone. Thus, the overall aim of the study was to gain a better understanding of side-effects of aeration and redox-based detoxification methods to provide guidance for the design of more efficient bioconversion processes.

## Results and discussion

The discovery of LPMO and its inclusion in enzyme preparations has opened up new possibilities to obtain increased yields of sugars and fermentation products from lignocellulose [17, 18]. It has also raised questions regarding suitable process conditions [24], such as the potential inclusion of an aeration step before the fermentation is initiated [27]. As a previous study of LPMO-supported saccharification suggested that there might be undesired side-effects of aeration [26], this was explored further in gas addition experiments with the liquid fraction of a slurry from pretreated softwood sawdust, followed by fermentation using the xylose-fermenting *S. cerevisiae* yeast strain CelluX™4. Fermentation experiments were performed analytically after gas addition steps and detoxification steps, to compare the fermentability after the different treatments.

### Series I: aeration vs. N_2_

Two pretreatment runs were made using similar feedstock and process conditions, and the corresponding liquid fractions are referred to as Pretreatment Liquid A (PL-A) and Pretreatment Liquid B (PL-B). In both PL-A and PL-B the three main sugars were glucose, mannose, and xylose (Table 1), all three of which are fermented by CelluX™4. As is typical for softwood hydrolysates due to the high content of galactoglucomannan [6], glucose and mannose were the most abundant sugar.

**Table 1:** Monosaccharides and pretreatment by-products in original PL-A and PL-B.^a^

Substance group/Analyte	PL-A	PL-B
*Monosaccharides (g L*^−1^*)*		
Arabinose	2.3 (0.1)	3.1 (0.1)
Galactose	4.8 (0.1)	5.6 (0.2)
Glucose	27.4 (0.1)	16.0 (0.1)
Mannose	23.2 (0.2)	21.9 (0.1)
Xylose	13.4 (0.2)	11.4 (0.3)
*Aliphatic aldehydes and alcohols (mM)*		
Acetaldehyde	0.184 (0.006)	0.196 (0.001)
Formaldehyde	9.7 (0.1)	10.7 (0.1)
Methanol	33.0 (2.2)	29.6 (1.7)
*Furan aldehydes (mM)*		
Furfural	22.3 (0.6)	15.2 (0.1)
HMF	18.5 (0.4)	10.3 (0.5)
*Aromatic substances and benzoquinone (μM)*		
Acetovanillone	3.4 (0.1)	ND
Benzoquinone	2.5 (0.1)	3.3 (0.2)
Coniferyl aldehyde	33.6 (0.6)	62.3 (0.8)
4-Hydroxybenzaldehyde	6.2 (0.3)	7.2 (0.2)
Vanillin	292 (7)	130 (2)

^a^The table shows mean values of technical triplicates with standard deviations in parentheses. Concentrations before addition of glucose to PL-B and before dilutions with water and other reaction constituents. ND, not detected. Sugars were determined using HPAEC-PAD and methanol using GC-FID. Furan aldehydes, aliphatic aldehydes, aromatic substances and benzoquinones were determined using UHPLC-ESI-QqQ-MS

Formaldehyde concentrations were quite high (~ 10 mM), whereas acetaldehyde concentrations were very low (~ 0.2 mM) (Table 1). Formaldehyde has been identified as the predominant inhibitor of yeast in pretreatment liquids of Norway spruce [28]. The low acetaldehyde content can potentially be attributed to the low acetyl group content of softwood, as acetyl groups could serve as precursors of acetaldehyde. With regard to furan aldehydes, the furfural concentrations exceeded those of HMF (Table 1).

Methanol formation during hydrothermal pretreatment has not received much attention previously, but the discovery of significant concentrations of formaldehyde [29] and the risk that the formaldehyde concentration would increase as the result of oxidation of methanol motivated elaborating a GC-FID (gas chromatography with flame ionization detection) method suitable for methanol quantitation in pretreatment liquids (subsection "Analysis of methanol using GC-FID"). The results (Table 1) show that the pretreatment liquids contained 30–33 mM (~ 1 g L^−1^) methanol. Thus, the methanol concentrations were roughly three times as high as those of formaldehyde and higher than those of furfural. The presence of methanol in pretreatment liquids is not surprising, since it is a known product in other types of thermal conversion of wood, in which it can form as a degradation product from methoxyl groups in lignin and hemicelluloses.

Other substances that were analyzed included three phenolic aldehydes (coniferyl aldehyde, *p*-hydroxybenzaldehyde, and vanillin), one phenolic ketone (acetovanillone), and benzoquinone (Table 1). Of these, acetovanillone, coniferyl aldehyde, and vanillin are typical degradation products of guaiacyl subunits in lignin, the predominant type of subunit in lignin from conifers [30], such as Norway spruce. Of the quantitated substances in this group, vanillin was, by far, the most abundant (Table 1). The aromatic substances that were quantitated are merely a few examples of the great variety of aromatic, including phenolic, substances that are found in lignocellulosic hydrolysates [9, 11, 31].

The differences between PL-A and PL-B can be due to differences between raw materials and differences in processing and storage. There are subtle differences that suggest that the pretreatment used for PL-A was somewhat harsher than the pretreatment used for PL-B, such as PL-A having higher glucose content than mannose content, and PL-A having higher content of furan aldehydes and vanillin than PL-B.

**Fig. 1:**
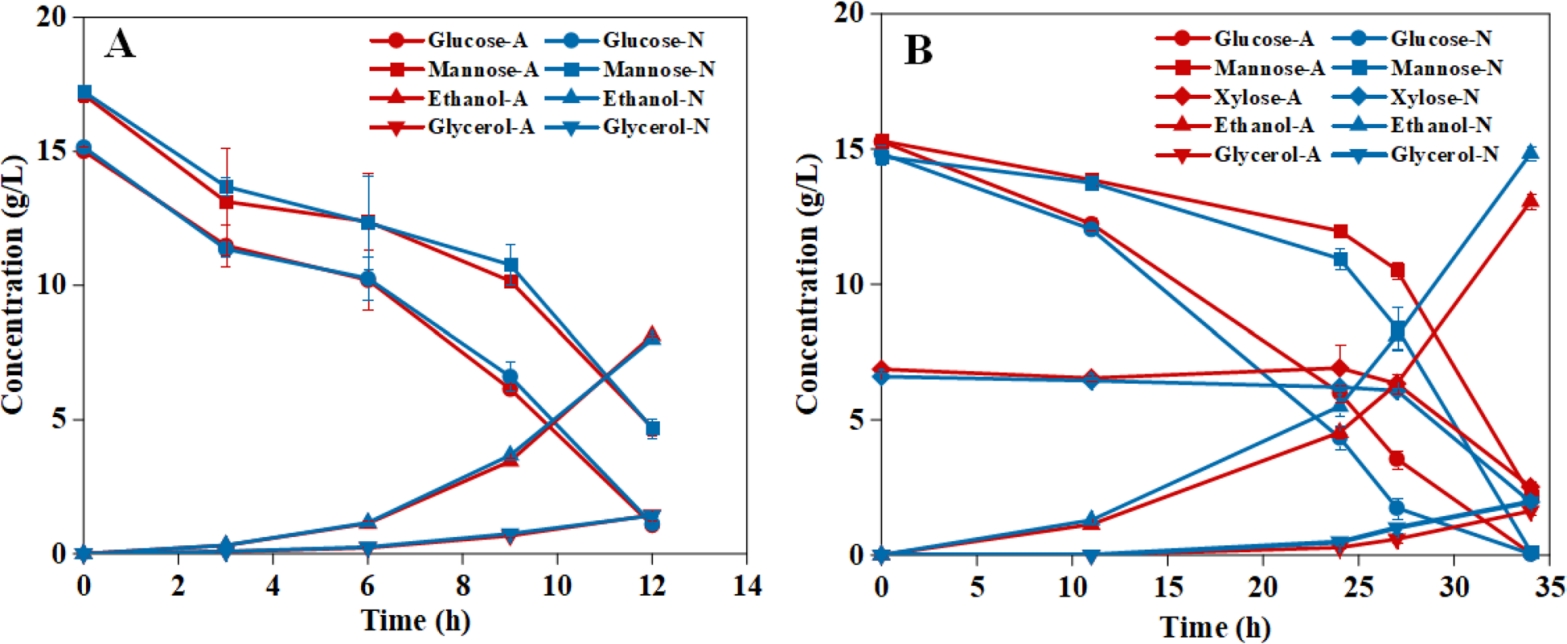
Concentrations of glucose ($\bigcirc$), mannose ($\square$), xylose ($\diamondsuit$), ethanol (∆), and glycerol (∇) in Series I (aeration vs. N_2_) for fermentations with (**A**) SCon (sugar control) medium and with (**B**) PL-A medium. Red lines and symbols, air ("A" in inset); blue lines and symbols, N_2_ ("N" in inset). For each symbol and time point, error bars show standard errors of triplicates (small errors may be concealed by symbols)

In Series I, aeration of PL was compared to a N_2_ control and to a sugar control. The sugar control was included to see if potential effects were dependent on components in the PL. The sugar control did not show any differences between aeration and N_2_, regardless of whether it was consumption of glucose and mannose or production of ethanol, glycerol, and acetate (Fig. 1A and Table 2). The experiments with PL showed significant differences, where fermentations after N_2_ addition consistently performed better than fermentations after aeration. Compared to fermentations after aeration, fermentations after N_2_ addition showed faster consumption of glucose (0.45 vs. 0.39 g L^−1^ h^−1^ after 27 h), mannose (0.21 vs. 0.13 g L^−1^ h^−1^ after 27 h), xylose (0.13 vs. 0.11 g L^−1^ h^−1^ after 34 h), and total fermentable sugar (0.76 vs. 0.61 g L^−1^ h^−1^ after 27 h), as well as faster production of ethanol (0.30 vs. 0.23 g L^−1^ h^−1^ after 27 h) and glycerol (0.22 vs. 0.20 g L^−1^ h^−1^ after 27 h) (Fig. 1B, Table 2). Also, the ethanol yield on consumed sugar (glucose, mannose, and xylose) was higher for fermentation after N_2_ addition than for fermentation after aeration (0.43 vs. 0.39 g g^−1^ after 34 h) (Table 2). The differences were statistically significant (p ≤ 0.01).

**Table 2:** Fermentation experiments with *S. cerevisiae* CelluX™4 in Series I-III.^a^

Experimental series and parameter^b^	GCR (g L^−1^ h^−1^)	MCR (g L^−1^ h^−1^)	XCR (g L^−1^ h^−1^)	FCR (g L^−1^ h^−1^)	EPR (g L^−1^ h^−1^)	GPR (g L^−1^ h^−1^)	APR (g L^−1^ h^−1^)	Y_CS_ (g g^−1^)
Ser. I SCon Air 12 h	1.08 (0.01)	0.94 (0.03)	NTD	2.02 (0.04)	0.68 (0.01)	0.12 (0.01)	0.02 (0.01)	0.34 (0.01)
Ser. I SCon N_2_ 12 h	1.07 (0.02)	0.94 (0.03)	NTD	2.02 (0.05)	0.66 (0.01)	0.12 (0.01)	0.02 (0.01)	0.33 (0.01)
Ser. I PL-A Air 27 h	0.39 (0.01)	0.13 (0.01)	0.01 (0.01)	0.61 (0.02)	0.23 (0.01)	0.20 (0.01)	NTD	0.38 (0.02)
Ser. I PL-A N_2_ 27 h	0.45 (0.01)	0.21 (0.03)	0.01 (0.01)	0.76 (0.04)	0.30 (0.02)	0.22 (0.01)	NTD	0.39 (0.01)
Ser. I PL-A Air 34 h	0.41 (0.01)	0.36 (0.03)	0.11 (0.01)	0.98 (0.03)	0.38 (0.01)	0.19 (0.01)	NTD	0.39 (0.01)
Ser. I PL-A N_2_ 34 h	0.41 (0.01)	0.41 (0.01)	0.13 (0.01)	1.02 (0.01)	0.44 (0.01)	0.20 (0.01)	NTD	0.43 (0.01)
Ser. IIa PL-A Air 29 h	0.36 (0.01)	0.11 (0.01)	0.02 (0.01)	0.54 (0.04)	0.15 (0.01)	NTD	NTD	0.29 (0.02)
Ser. IIa PL-A N_2_ 29 h	0.04 (0.01)	0.01 (0.01)	0.02 (0.01)	0.17 (0.01)	0.01 (0.01)	NTD	NTD	0.06 (0.01)
Ser. IIa PL-A Air 34 h	0.41 (0.01)	0.33 (0.02)	0.05 (0.01)	0.86 (0.03)	0.28 (0.05)	NTD	NTD	0.32 (0.01)
Ser. IIa PL-A N_2_ 34 h	0.02 (0.01)	0.01 (0.01)	0.01 (0.01)	0.10 (0.01)	0.01 (0.01)	NTD	NTD	0.09 (0.01)
Ser. IIb PL-A Air 29 h	0.46 (0.03)	0.26 (0.01)	0.03 (0.01)	0.84 (0.02)	0.25 (0.01)	NTD	NTD	0.30 (0.01)
Ser. IIb PL-A N_2_ 29 h	0.03 (0.01)	0.01 (0.01)	0.01 (0.01)	0.09 (0.01)	0.02 (0.01)	NTD	NTD	0.16 (0.02)
Ser. IIb PL-A Air 36 h	0.39 (0.03)	0.39 (0.01)	0.14 (0.01)	0.99 (0.01)	0.34 (0.01)	NTD	NTD	0.34 (0.01)
Ser. IIb PL-A N_2_ 36 h	0.03 (0.01)	0.01 (0.01)	0.01 (0.01)	0.10 (0.01)	0.01 (0.01)	NTD	NTD	0.14 (0.02)
Ser. IIIa PL-B Air 12 h	0.50 (0.01)	0.15 (0.03)	0.04 (0.02)	0.69 (0.06)	0.20 (0.01)	NTD	NTD	0.30 (0.02)
Ser. IIIa PL-B N_2_ 12 h	0.33 (0.03)	0.10 (0.04)	0.04 (0.02)	0.47 (0.09)	0.14 (0.01)	NTD	NTD	0.30 (0.02)
Ser. IIIa PL-B Air 18 h	0.75 (0.01)	0.53 (0.04)	0.07 (0.01)	1.36 (0.06)	0.47 (0.01)	NTD	NTD	0.35 (0.01)
Ser. IIIa PL-B N_2_ 18 h	0.50 (0.02)	0.16 (0.02)	0.04 (0.01)	0.69 (0.04)	0.24 (0.01)	NTD	NTD	0.34 (0.01)
Ser. IIIb SCon Air 8 h	0.91 (0.03)	0.40 (0.05)	NTD	1.31 (0.08)	0.50 (0.01)	NTD	NTD	0.38 (0.02)
Ser. IIIb SCon N_2_ 8 h	0.92 (0.03)	0.40 (0.05)	NTD	1.32 (0.07)	0.52 (0.01)	NTD	NTD	0.40 (0.02)
Ser. IIIb SCon Air 12 h	1.07 (0.01)	1.09 (0.1)	NTD	2.15 (0.01)	0.89 (0.01)	NTD	NTD	0.41 (0.02)
Ser. IIIb SCon N_2_ 12 h	1.07 (0.01)	1.09 (0.1)	NTD	2.16 (0.01)	0.89 (0.01)	NTD	NTD	0.41 (0.02)

^a^GCR, glucose consumption rate; MCR, mannose consumption rate; XCR, xylose consumption rate; FCR, fermentable sugar (glucose and mannose, and for PL also xylose) consumption rate; EPR, ethanol production rate; GPR, glycerol production rate; APR, acetate production rate; Y_CS_ ethanol yield on consumed sugar (glucose + mannose + xylose). NTD, not determined. ^b^PL-A, Pretreatment liquid A; PL-B, Pretreatment liquid B; SCon, sugar control; Ser. I, Aeration vs. N_2_; Ser. IIa, Sulfite before aeration; Ser. IIb, Sulfite after aeration; Ser. IIIa, Pretreatment liquid spiked with hydroquinone before aeration; Ser. IIIb, Sugar control spiked with hydroquinone before aeration

Potential changes in the concentrations of pretreatment by-products in the Series I experiment were analyzed using HPLC-RID (high-performance liquid chromatography with refractive index detection), GC-FID, and UHPLC-ESI-QqQ-MS (ultra-high-performance liquid chromatography-electrospray ionization-triple quadrupole-mass spectrometry) (Table 3). Acetic acid concentrations remained stable at around 60 mM, unaffected by either aeration or N_2_. There was a significant (p ≤ 0.01) decrease in the concentrations of acetaldehyde. Formaldehyde increased slightly after aeration and decreased slightly after N_2_ treatment, but differences between air and N_2_ were small (4%) and not statistically significant. Methanol concentrations dropped dramatically (~ 30%) for both aeration and N_2_, slightly more for aerated medium, although the difference compared to N_2_ was not statistically significant (p >  > 0.1). The most probable reason for this is that the gas flow caused evaporation of methanol.

**Table 3:** Analysis of pretreatment by-products in Series I and II (PL-A).^a,b^

Substance group/Analyte	Before gas addition	Series I Air	Series I N_2_	Series IIa Air	Series IIb Air	Series II N_2_
*Aliphatic acids (mM)*						
Acetic acid	58.7 (0.8)	62.2 (1.2)	62.1 (0.9)	59.5 (0.1)	59.2 (0.1)	59.1 (0.2)
*Aliphatic aldehydes and alcohols (mM)*						
Acetaldehyde	0.139 (0.002)	0.109 (0.003)	0.108 (0.007)	0.103 (0.001)	0.110 (0.003)	0.105 (0.003)
Formaldehyde	5.3 (0.1)	5.4 (0.2)	5.2 (0.1)	1.3 (0.1)	5.6 (0.2)	5.3 (0.1)
Methanol	18.7 (1.4)	12.7 (1.0)	13.2 (0.7)	NTD	NTD	NTD
*Furan aldehydes (mM)*						
Furfural	13.8 (0.4)	9.4 (0.2)	10.6 (0.1)	10.9 (0.1)	12.1 (0.3)	8.6 (0.1)
HMF	10.6 (0.2)	7.5 (0.1)	9.3 (0.2)	11.8 (0.1)	13.0 (0.1)	7.3 (0.1)
*Aromatic substances and benzoquinone (μM)*						
Acetovanillone	3.6 (0.3)	3.0 (0.1)	3.0 (0.2)	3.5 (0.2)	3.3 (0.1)	2.8 (0.2)
Benzoquinone	5.5 (0.3)	5.9 (0.4)	7.1 (0.3)	2.3 (0.2)	4.4 (0.3)	4.5 (0.2)
Coniferyl aldehyde	24.5 (0.5)	23.3 (1.0)	25.1 (0.6)	15.1 (0.5)	26.2 (0.7)	25.3 (0.6)
4-Hydroxybenzaldehyde	7.2 (0.1)	6.2 (0.1)	5.6 (0.2)	6.6 (0.1)	5.9 (0.1)	5.3 (0.1)
Vanillin	165 (4)	159 (2)	141 (3)	162 (3)	160 (4)	136 (2)

^a^PL-A (1.75 times dilution). Mean values of technical triplicates with standard deviations in parentheses. Acetic acid was analyzed using HPLC-RID, methanol using GC-FID, and all other substances using UHPLC-ESI-QqQ-MS. "Air" and "N_2_" refer to samples taken after 24 h of gas addition. NTD, not determined. ^b^Series I, Aeration vs. N_2_; Series IIa, Sulfite before aeration; Series IIb, Sulfite after aeration; Series II N_2_, no sulfite

The concentrations of furan aldehydes decreased significantly (p ≤ 0.01) during both aeration (29–32%) and N_2_ treatment (12–23%). The decrease of furfural was somewhat higher than that of HMF, as expected, since furfural is more volatile and therefore easier evaporated by gas addition than HMF.

Among aromatic substances, there were minor but significant decreases in the concentration of vanillin for both air (p ≤ 0.05) and N_2_ (p ≤ 0.01). As vanillin is volatile, the decrease can be attributed to evaporation during gas addition.

### Series II: detoxification with sulfite

Detoxification with sulfur oxyanions, such as sulfite and dithionite, has benefits for alleviating both enzyme and fermentation inhibition, whereas other reducing agents, such as borohydride, have shown benefits only with respect to fermentation inhibitors. This is explained by different abilities of the reagents to hydrophilize and reduce their targets [10]. To alleviate enzyme inhibition, it would therefore be best to add detoxification agents such as sulfite or dithionite at an early stage, already when the enzymatic saccharification is initiated.

To investigate whether the detoxification effect of sulfite on fermentation inhibitors would be affected by aeration, experiments were conducted in which detoxification was carried out either before (Series IIa) or after (Series IIb) aeration. In both Series IIa and IIb, the detoxified reaction mixtures (IIa PL-A Air and IIb PL-A Air) fermented much better than the corresponding controls without any sulfite added (IIa PL-A N_2_ and IIb PL-A N_2_) (Table 2). This trend was apparent for all parameters, although xylose utilization lagged somewhat behind that of glucose and mannose, resulting in low initial xylose consumption rates for all fermentations. Thus, sulfite detoxification before aeration emerges as a viable option that could alleviate inhibition of both enzymes and the fermenting microbe.

The sulfite detoxification effect is apparent with regard to formaldehyde, which was reduced to only 1.3 mM for Series IIa Air compared to 5.6 mM for Series IIb Air, for which the sample for chemical analysis was taken after aeration but before the additions of sulfite and yeast (Table 3). Similarly, the coniferyl aldehyde concentration was only 15 μM in Series IIa Air but 26 μM in Series IIb Air. As for Series I, acetaldehyde decreased for all treatments. In contrast, aeration (Series IIb Air) resulted in a small but significant increase in the formaldehyde concentration (to 5.6 mM, p ≤ 0.05), whereas it stayed at 5.3 mM in mixtures with N_2_ addition.

### Series III: spiking with hydroquinone

Among inhibitors in softwood hydrolysates, benzoquinone was found to exhibit the highest molar toxicity [28]. Therefore, even though its concentrations are generally very low [32], benzoquinone is a substance of interest to analyze. Lignocellulosic hydrolysates may also contain hydroquinone [31], which has, however, a very low toxicity and which is relatively harmless to yeast [33]. However, during an oxidation harmless substances, such as hydroquinone, can potentially be oxidized to highly toxic substances, such as benzoquinone. So, on the one hand, hydroquinone could serve as a precursor of highly toxic benzoquinone, but, on the other hand, hydroquinone could potentially serve as a reducing agent and alleviate inhibition. Neither Series I nor Series II suggested an increase in benzoquinone concentrations as a result of aeration (Table 2), but concentrations were generally very low, ~ 5 μM, which is below the lowest toxic concentration tested by Stagge et al. [32], which was 2 mg L^−1^, i.e., ~ 20 μM. Therefore, an experimental series, Series III, was conducted in which the PL was spiked with hydroquinone before aeration.

Spiking with hydroquinone before treatment of PL with air (Ser. IIIa PL-B Air) consistently resulted in improved fermentation rates compared to the N_2_-treated control (Ser. IIIa PL-B N_2_). This includes consumption of glucose, mannose, xylose, and total fermentable sugar, as well as production of ethanol (Table 2). The ethanol yield on consumed sugar was, however, similar. In contrast, spiking with hydroquinone before treatment of the sugar control with air (Ser. IIIb SCon Air) had no effect compared to the N_2_-treated control (Ser. IIIb SCon N_2_). Thus, the effect of spiking with hydroquinone was positive rather than negative, and only with the inhibitor-containing PL rather than with the inhibitor-less sugar control. The result shows that addition of hydroquinone works as a detoxification method, similarly to the addition of other reducing agents, such as sulfur oxyanions and borohydride.

Spiking with hydroquinone interfered with the analysis of benzoquinone (Series IIIa Air in Table 4). Again, both aeration and N_2_ addition resulted in a decrease in acetaldehyde, whereas the formaldehyde concentration was slightly enhanced in the aerated reaction but not in the N_2_ reaction. Although the formaldehyde increase was small (0.3 mM), this was the third experimental series pointing in the same direction: Both aeration and N_2_ caused a significant (p ≤ 0.01) decrease (in the range 21%-50%) of the concentration of acetaldehyde, while aeration caused a very small but measurable increase (in the range 2–6%) in the concentration of formaldehyde, an increase that did not occur with N_2_ addition. The increase of formaldehyde in the aerated reaction was statistically significant (p ≤ 0.05).

**Table 4:** Analysis of pretreatment by-products in Series IIIa (PL-B).^a,b^

Substance group/Analyte	Before gas addition	Series IIIa Air	Series IIIa N_2_
*Aliphatic acids (mM)*			
Acetic acid	44.0 (0.2)	45.2 (0.2)	44.9 (0.1)
*Aliphatic aldehydes and alcohols (mM)*			
Acetaldehyde	0.120 (0.002)	0.065 (0.002)	0.060 (0.002)
Formaldehyde	5.5 (0.2)	5.8 (0.1)	5.4 (0.1)
Methanol	14.1 (1.1)	9.9 (0.4)	10.4 (0.3)
*Furan aldehydes (mM)*			
Furfural	7.4 (0.1)	6.8 (0.2)	7.6 (0.2)
HMF	4.7 (0.1)	5.8 (0.1)	7.0 (0.1)
*Aromatic substances and benzoquinone (μM)*			
Acetovanillone	ND	ND	ND
Benzoquinone	5.2 (0.3)	NTD	5.2 (0.5)
Coniferyl aldehyde	36.6 (1.8)	38.3 (1.8)	40.2 (1.0)
4-Hydroxybenzaldehyde	5.4 (0.2)	5.2 (0.1)	4.2 (0.1)
Vanillin	71.0 (2.3)	71.1 (0.6)	60.0 (1.0)

^a^PL-B (twofold dilution). Mean values of technical triplicates. Standard deviations are shown in parentheses. Acetic acid was analyzed using HPLC-RID, methanol using GC-FID, and all other substances using UHPLC-ESI-QqQ-MS. "Air" and "N_2_" refer to samples taken after 24 h of gas addition. ND, not detected. NTD, not determined. ^b^Series IIIa, Pretreatment liquid spiked with hydroquinone before aeration

Small increases in formaldehyde concentrations may still significantly impact fermentability. Cavka et al. [29] found a clear inhibitory effect already at 1 mM, and concentrations of around 5 mM were sufficient to almost completely inhibit fermentation with *S. cerevisiae*. That also explains why it was necessary to dilute the PLs in this study to get fermentation results within a reasonable time frame.

Analyses of Series IIIa showed a decrease (26–30%) in the concentration of methanol after gas addition (Table 4). The decrease after aeration was again slightly higher than the decrease after N_2_ addition, an observation that triggered Series VI, in which potential oxidation of methanol to formaldehyde was further examined.

### Series IV: addition of hydroquinone and laccase to pretreatment liquid

Although aeration may cause some oxidation reactions in lignocellulosic hydrolysates, the use of catalysts, such as the phenol oxidase laccase, can greatly enhance oxidation effects. Treatment with laccase has been found to improve the fermentability of phenol-rich hydrolysates, such as those from willow and olive tree pruning [12, 14]. Even if spiking with hydroquinone was not negative as such (Series III), there is a potential risk with laccase treatment causing oxidation of hydroquinone to toxic benzoquinone. An experimental series (Series IV) was therefore conducted using shake flasks to assess potential effects of addition of hydroquinone and laccase to PL-based medium.

**Fig. 2:**
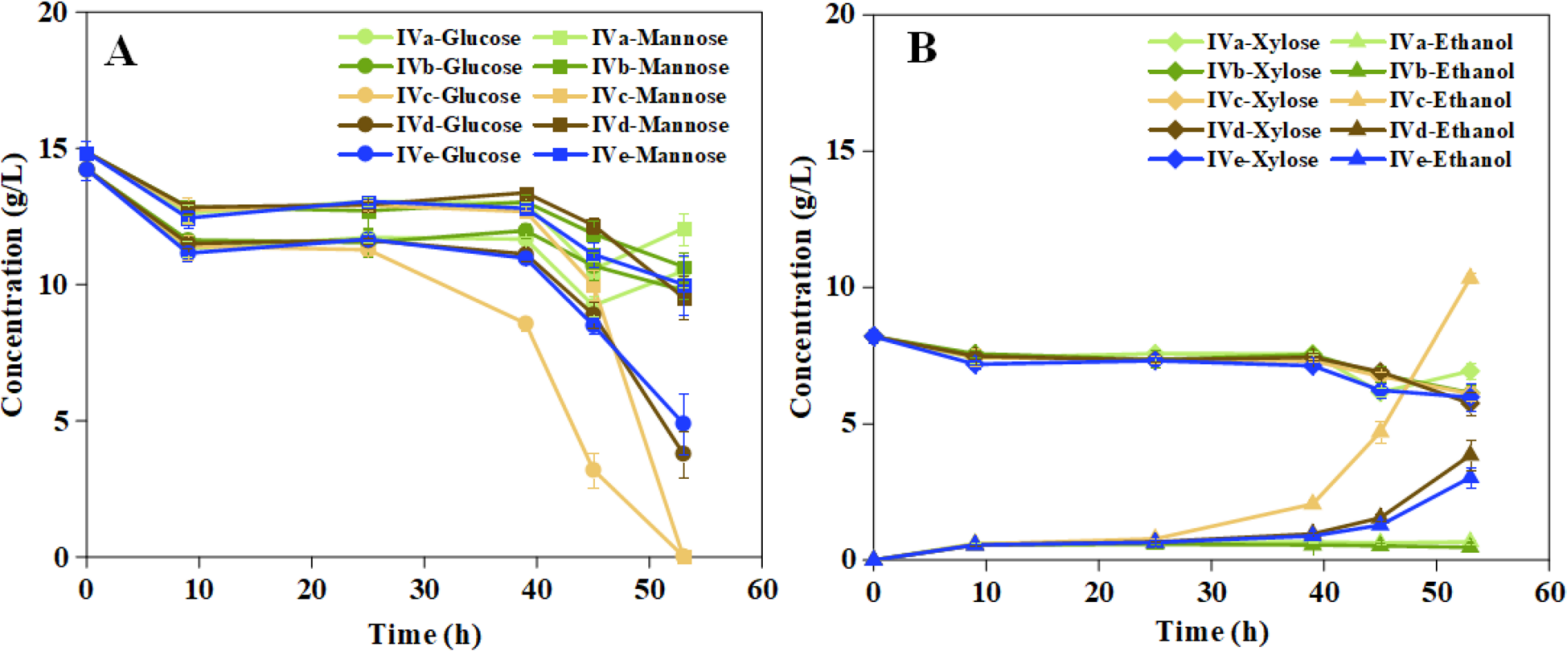
Fermentations in Series IV (addition of hydroquinone, HQ, and/or laccase, LCC, to pretreatment liquid PL-B) showing (**A**) concentrations of glucose ($\bigcirc$) and mannose ($\square$), and (**B**) concentrations of xylose ($\diamondsuit$) and ethanol (∆). Lines and symbols: Light green, IVa (LCC); dark green, IVb (LCC + HQ); yellow, IVc (HQ-B, with HQ added before incubation); brown, IVd (HQ-A, with HQ added after incubation); blue, IVe (control, no LCC or HQ). For each symbol and time point, error bars show standard errors of triplicates (small errors may be concealed by symbols)

A comparison between cultures with hydroquinone added before incubation (Ser. IVc HQ-B) and control cultures without additions (Ser. IVe Control) confirmed the results from Series IIIa, namely that addition of 1 mM hydroquinone was highly beneficial and served as a detoxification method. Early addition of hydroquinone promoted the consumption rates of glucose (0.27 vs. 0.18 g L^−1^ h^−1^), mannose (0.28 vs. 0.09 g L^−1^ h^−1^), and fermentable sugar (0.59 vs. 0.31 g L^−1^ h^−1^), as well as the ethanol productivity (0.20 vs. 0.06 g L^−1^ h^−1^) and the ethanol yield on consumed sugar (0.33 vs. 0.19 g g^−1^) (Table 5). Addition of hydroquinone after the treatment (Ser. IVd HQ-A) increased the performance compared to the control without additions (Ser. IVe Control) a little, but much less than when the hydroquinone was added before the treatment (Ser. IVd HQ-B). In contrast, the mixtures that contained laccase (Ser. IVa LCC and Ser. IVb LCC + HQ) performed worse than the control without additions (Ser. IVe Control) (Fig. 2, Table 5).

**Table 5:** Fermentation experiments with *S. cerevisiae* CelluX™4 in Series IV.^a,b^

Experimental series and parameter	GCR (g L^−1^ h^−1^)	MCR (g L^−1^ h^−1^)	XCR (g L^−1^ h^−1^)	FCR (g L^−1^ h^−1^)	EPR (g L^−1^ h^−1^)	Y_CS_ (g g^−1^)
Ser. IVa LCC 53 h	0.08 (0.01)	0.06 (0.01)	0.02 (0.01)	0.16 (0.01)	0.01 (0.01)	0.09 (0.02)
Ser. IVb LCC + HQ 53 h	0.09 (0.01)	0.08 (0.01)	0.04 (0.01)	0.19 (0.03)	0.01 (0.01)	0.04 (0.01)
Ser. IVc HQ-B 53 h	0.27 (0.01)	0.28 (0.01)	0.04 (0.01)	0.59 (0.01)	0.20 (0.01)	0.33 (0.01)
Ser. IVd HQ-A 53 h	0.20 (0.02)	0.10 (0.02)	0.05 (0.01)	0.34 (0.03)	0.07 (0.01)	0.21 (0.03)
Ser. IVe Control 53 h	0.18 (0.02)	0.09 (0.02)	0.04 (0.01)	0.31 (0.05)	0.06 (0.01)	0.19 (0.02)

^a^GCR, glucose consumption rate; MCR, mannose consumption rate; XCR, xylose consumption rate; FCR,

fermentable sugar (glucose, mannose, and xylose) consumption rate; EPR, ethanol production rate; Y_CS_ ethanol

yield on consumed sugar (glucose + mannose + xylose). ^b^Series IV, Addition of hydroquinone and/or laccase to

pretreatment liquid PL-B. LCC, laccase; HQ, hydroquinone; HQ-B, HQ added before incubation; HQ-A, HQ added

after incubation; Control, no LCC or HQ

After the 12 h treatment and before inoculation, the total phenolic content was 1.0 ± 0.1 g L^−1^ in PL with laccase, and 2.3 ± 0.1 g L^−1^ in PL without additions. Although this clear decrease would typically suggest a detoxification effect, the treatment resulted in increased toxicity, indicating that some reaction products must have been more toxic than the original substrates. As the PL is a complex mixture of many different substances, including phenolic substances [9, 11, 31] that could be substrates for laccase [13], an experimental series (Series V) was performed with a simpler system based on sugar control medium.

### Series V: addition of hydroquinone and laccase to sugar control

As there are a lot of potential substrates for laccase in hydrolysates, an experiment (Series V) was done using a simplified system consisting of sugar control medium without any PL, but with hydroquinone.

**Fig. 3:**
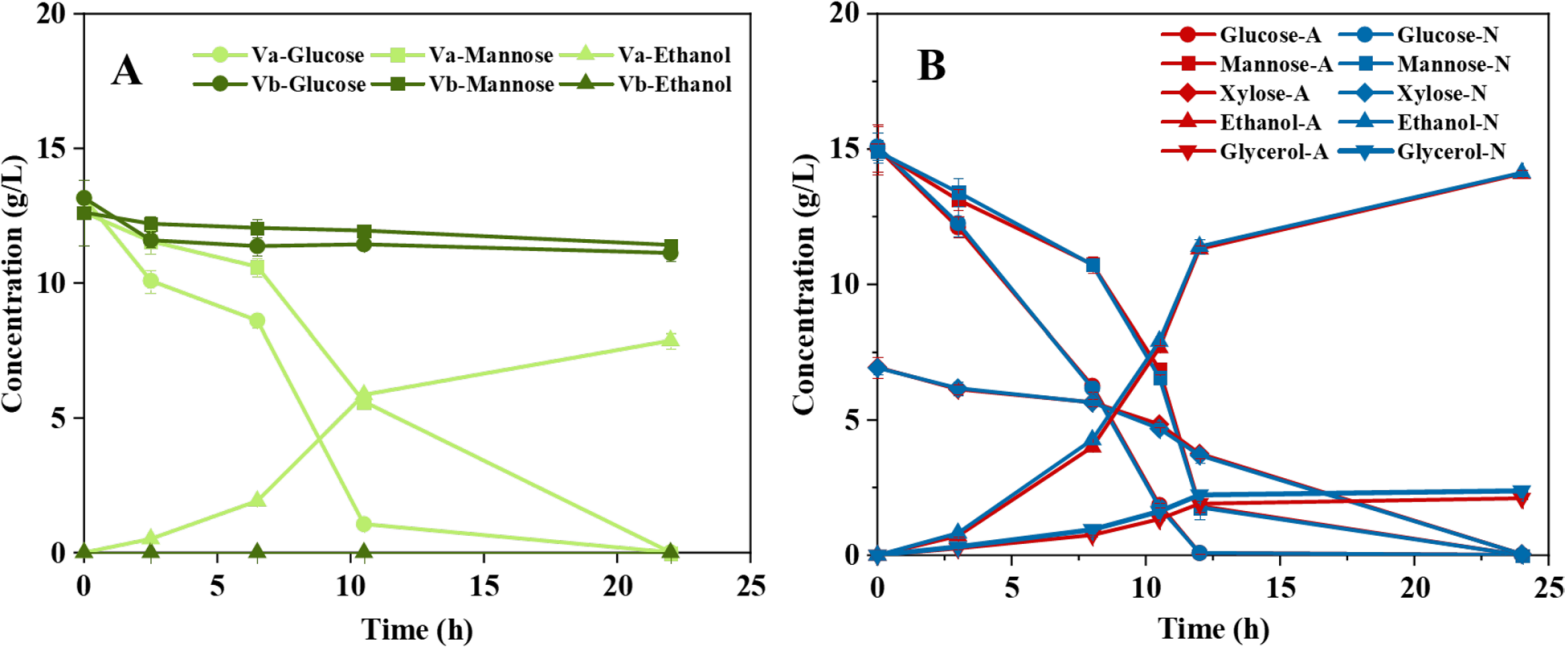
(A) Concentrations of glucose ($\bigcirc$), mannose ($\square$), and ethanol (∆) in Series V fermentations (addition of hydroquinone, HQ, and laccase, LCC, to sugar control, SCon). Light green lines and symbols, with laccase Va (LCC); dark green lines and symbols, with both laccase and hydroquinone Vb (LCC + HQ). (B) Concentrations of glucose ($\bigcirc$), mannose ($\square$), xylose ($\diamondsuit$), ethanol (∆), and glycerol (∇) in series VI fermentations. Red lines and symbols, air; blue lines and symbols, N_2_. For each symbol and time point, error bars show standard errors of triplicates (small errors may be concealed by symbols)

When only laccase was added, the fermentation proceeded rapidly. After 10.5 h, almost all glucose and about half of the mannose were consumed, with substantial ethanol production (5.9 g L^−1^) (Fig. 3A). In contrast, simultaneous addition of both laccase and hydroquinone to the medium led to almost no fermentative activity. Even after 22 h of fermentation, sugar consumption was minimal and ethanol was hardly detectable (< 0.01 g L^−1^). After 10.5 h fermentation with laccase alone, glucose and mannose consumption rates were 1.15 and 0.67 g L^−1^ h^−1^, respectively, with an ethanol production rate of 0.56 g L^−1^ h^−1^ and a yield of 0.31 g g^−1^ on consumed sugar. With both laccase and hydroquinone, glucose and mannose consumption rates dropped to 0.17 and 0.07 g L^−1^ h^−1^, respectively, with an ethanol production rate below 0.01 g L^−1^ h^−1^ and a yield on consumed sugar of only 0.04 g g^−1^.

It is obvious from the result that laccase catalyzed an oxidation of hydroquinone to a substance with much higher toxicity, presumably benzoquinone. Also, as the initial concentration of hydroquinone was relatively low (1 mM corresponding to 0.1 g L^−1^), it was not inhibitory from the beginning. Larsson et al. [33] found that hydroquinone concentrations as high as 1 g L^−1^ did not have any toxic effect on *S. cerevisiae* yeast, except a minor negative effect on biomass yield.

### Series VI: assessing the impact of methanol

As analyses of PL-A and PL-B media had shown minor differences between aerated and N_2_-treated samples with respect to methanol and formaldehyde (Tables 3 and 4), a separate experiment was conducted to assess the potential impact of methanol on the fermentability under aeration. This experimental series was also used to assess whether aeration caused any measurable oxidation of methanol to formaldehyde, as judged by analysis of potential formaldehyde in the medium after aeration.

With 0.50 g L^−1^ methanol in the medium (a concentration that was similar to the concentrations in cultivations using PL-A and PL-B media), there was no difference between aerated cultures and N_2_ control cultures, neither with respect to sugar consumption (glucose, mannose, xylose) nor to product formation (ethanol, glycerol) (Fig. 3B). The possibility that small quantities of formaldehyde were formed in aerated cultures was nevertheless investigated using UHPLC-ESI-QqQ-MS, but no formaldehyde was detectable. Thus, the decrease in methanol concentrations detected in experiments with PL-A and PL-B was attributed to evaporation rather than to oxidation to formaldehyde.

## Conclusions

Although aeration during enzymatic saccharification of pretreated lignocellulose is beneficial for supporting LPMO, it may, at least in some cases, have undesired side-effects causing more severe inhibition. Compared to N_2_ controls, aeration consistently resulted in a small but measurable increase of the concentration of formaldehyde. Whether that is a problem or not would depend on other factors. With regard to volatile inhibitors, such as furfural, aeration had a positive effect by reducing the concentrations due to evaporation. Laccase treatment can have both positive and negative effects depending on the composition and concentrations of inhibitors in the medium. Detoxification with sulfite is a possibility to alleviate increased inhibition problems caused by aeration.

## Methods

### Pretreatment of biomass

The lignocellulosic slurries used for preparation of pretreatment liquids were produced in the Biorefinery Demo Plant (Örnsköldsvik, Sweden) by Sekab E-Technology. The production process involved continuous steam explosion with sulfur dioxide serving as catalyst. The feedstock was sawdust from debarked Norway spruce (*Picea abies*). The sulfur dioxide loading rate was 0.3 kg h^−1^, the processing temperature was 195°C (corresponding to an absolute pressure of around 14 bar), and the residence time was approx. 12 min. After cooling, the pH of the slurries was measured and found to be 1.6–1.7. The approx. total solids (TS) content (w/w) of the corresponding slurries was 25% for PL-A and 23% for PL-B.

Before further experiments, the solid and liquid phases of the slurries were separated using centrifugation. Potential residual particles in the liquid phase left after centrifugation were removed by sequential filtration through sterile filters, first with pore size 0.45 µm and then with pore size 0.2 µm (PES, Nalgene™ Rapid-Flow™, Thermo Fisher Scientific, Waltham, MA).

### Gas addition using air and nitrogen gas

Pretreatment liquids and control solutions were treated using aeration to mimic conditions used to promote LPMO-supported saccharification of biomass. Nitrogen gas was used for comparison with aerated solutions. When pretreatment liquid was treated by gas addition, the pH was first adjusted to 5.5 using a 10 M aqueous solution of NaOH.

Gas addition was performed using an in-house laboratory system consisting of six parallel 55 mL reaction bottles partially immersed in a water bath with thermostatic temperature control and positioned on top of a multipoint magnetic stirrer (Cimarec, Thermo Fisher Scientific). Two three-way gas distributors (one for air and one for N_2_) were used to divide the gas into three strands, each connected to a flow meter (Model F65, Porter Instrument Division, Parker Hannifin Corporation, Hatfield, PA) using Polyamide 12 tubing with an outer diameter of 6 mm. Three of the bottles received a continuous supply of air and the other three received a continuous supply of N_2_ (99.996% purity). Gas was supplied through a sterile needle (0.9 × 70 mm), while a shorter needle (0.8 × 50 mm) was inserted into the rubber cap of the flask to vent gas. The gas flow rate was maintained at 0.2 vvm (gas volume per reaction mixture volume per min). Each bottle was equipped with a cylindrical magnetic stirrer bar (10 × 3 mm). The stirring speed was maintained at 130 rpm. The duration of the gas addition was 24 h and the temperature was 30 °C. Experiments were conducted in triplicate (three bottles with air and three with N_2_ in each experimental series).

### Fermentation experiments

Fermentation experiments were conducted in six series (I-VI), described in more detail below. Prior to all fermentations, the pH of pretreatment liquids was checked and, if needed, adjusted to 5.5 using the 10 M sodium hydroxide solution. Pretreatment liquids were diluted with ultra-pure water to achieve suitable experimental conditions (1.75-fold dilution for PL-A and a 2.0-fold dilution for PL-B). Glucose was added to PL-B so that it contained the same glucose level as PL-A.

All fermentation experiments were performed using *S. cerevisiae* CelluX™4 (Leaf, Marcq-en-Barœul Cedex, France). The dry yeast was reconditioned by incubation in autoclaved water at 30 °C and continuous shaking at 100 rpm for 30 min. In typical experiments with PL-A and PL-B, the yeast inoculum was 1.0 g L^−1^ and 2.0 g L^−1^, respectively.

If not otherwise stated, the fermentation experiments were conducted in 55 mL serum bottles sealed with rubber caps and equipped with cannulas to release carbon dioxide formed during fermentation. Each bottle contained 30 mL reaction mixture including nutrient solution [1.5 mL consisting of 3.75 g L^−1^ MgSO_4_ • 7 H_2_O, 238.2 g L^−1^ NaH_2_PO_4_ • H_2_O, 75 g L^−1^ (NH_4_)_2_HPO_4_, and 150 g L^−1^ yeast extract] and yeast inoculum (0.6 mL to reach 1 g L^−1^, or 1.2 mL to reach 2 g L^−1^). Fermentations were carried out in a shaking incubator at 30 °C.

For all fermentation series, samples (0.5 mL) for chemical analysis were withdrawn at different time points using a sterile syringe. The collected samples were centrifugated and the supernatant was kept in the freezer until analysis. The glucose content in reaction mixtures was analyzed using high-performance anion-exchange chromatography (HPAEC) as detailed in the subsection "Analysis of monosaccharides using HPAEC-PAD." Ethanol was analyzed using HPLC-RID as detailed in the subsection "Analysis of ethanol, glycerol and acetate using HPLC-RID".

### Series I: effects of gas addition to pretreatment liquid

The purpose of the experiment was to detect potential effects of aeration, using N_2_ gas for comparison. To obtain a convenient time course for carrying out the experiment, pretreatment Liquid A (PL-A) was diluted 1.75 times using ultra-pure water. The initial composition of PL-A, before dilution, is shown in Table 1. A sugar control (SCon), consisting of a mixture of glucose and mannose (about 15 g L^−1^ of each) without any PL (but with the nutrient solution mentioned above), was also included in the experiment.

### Series IIa and IIb: detoxification with sodium sulfite

Two series of detoxification experiments (IIa and IIb) were conducted with 1.75 times diluted PL-A with sodium sulfite added as powder to a final concentration of 10 mM in bottles subjected to aeration. In IIa the sodium sulfite was added before aeration, whereas in IIb it was added after aeration. The bottles subjected to N_2_ contained no sodium sulfite, and were only used as a reference.

### Series III: addition of hydroquinone before aeration

In Series IIIa, a freshly prepared aqueous solution of hydroquinone was added to bottles with two-times diluted PL-B (Table 1) to reach a final concentration of 1 mM before aeration. Medium treated with N₂, without hydroquinone, was prepared and fermented for comparison. Additionally, a comparative fermentation experiment (Series IIIb) was carried out by adding hydroquinone to a buffered (40 mM sodium acetate buffer pH 5.5) sugar solution (about 15 g L^−1^ of each of glucose and mannose) (including the nutrient solution mentioned above). The detailed procedure for this experiment was otherwise identical to the one performed using PL-B, as described above.

### Series IV: addition of hydroquinone and/or laccase to pretreatment liquid

An experiment with hydroquinone (HQ) and/or laccase (LCC) in two-times diluted PL-B medium was conducted without prior gas addition using 100 mL Erlenmeyer flasks with 30 mL medium. The flasks were placed in a shaking incubator (160 rpm) and were equipped with cotton plugs to allow exchange of air to drive laccase-catalyzed reactions. When included, the concentrations of laccase and hydroquinone added to the reaction mixtures were 1 μM and 1 mM, respectively. The laccase was purified in-house from cultures of the white-rot fungus *Trametes versicolor*. The laccase preparation that was used for the experiment was purified to homogeneity, as judged from SDS-PAGE and staining with Coomassie Brilliant Blue. It was blue in color, had an activity of 69,000 U L^−1^ (ABTS assay), and the protein content was 6.7 mg mL^−1^. There was a 12 h initial incubation period (30 °C with shaking) before yeast addition. In some cases, hydroquinone was added before the 12 h initial incubation period and in some cases after (see further details below). When included, laccase was always added before the 12 h initial incubation period.

Five sets of conditions were included, each in triplicate (i.e., 15 flasks in total): IVa, laccase (LCC); IVb, laccase and hydroquinone (LCC + HQ) added before the initial incubation period; IVc, hydroquinone added before ("B") the initial incubation period (HQ-B); IVd, hydroquinone added after ("A") the initial incubation period (HQ-A); IVe, no addition of laccase or hydroquinone.

### Series V: addition to buffer of hydroquinone (HQ) and/or laccase

Additionally, a series comparative fermentation experiments in Erlenmeyer flasks was conducted as described for Series IV, but utilizing a medium consisting of a buffered (40 mM sodium acetate pH 5.5) sugar solution (about 15 g L^−1^ of each of glucose and mannose) (including the nutrient solution mentioned above). The concentrations of hydroquinone and laccase were the same as in Series IV. Two sets of conditions were included: Va, including only laccase (LCC); Vb, including both laccase and hydroquinone (LCC + HQ). Both laccase and hydroquinone were added before the initial 12 h incubation period.

### Series VI: assessing the impact of methanol

Before gas addition, methanol was added to the medium at a concentration of 0.50 g L^−1^ (16 mM). The medium was a buffered (40 mM sodium acetate, pH 5.5) aqueous solution containing a sugar mixture consisting of 15 g L^−1^ glucose, 15 g L^−1^ mannose, and 7 g L^−1^ xylose, also containing the previously described nutrient solution in the same concentration as before. Other experimental procedures were consistent with those described above for the PL-B fermentation experiments.

### Analysis of reaction mixtures

#### Analysis of monosaccharides using HPAEC-PAD

As HPLC-RID does not offer full separation of all major monosaccharides in lignocellulosic hydrolysates, the monosaccharides were instead analyzed using high-performance anion-exchange chromatography with pulsed amperometric detection (HPAEC-PAD). The HPAEC-PAD system (Dionex ICS-6000) included a guard column (4 × 50 mm) paired with a CarboPac PA1 separation column (4 × 250 mm), and an electrochemical detector (all from Thermo Fisher Scientific). The column temperature was maintained at 30 °C. The samples were diluted with ultra-pure water and filtered (0.20 µm nylon membrane filters from Merck Millipore Ltd., Cork, Ireland) before analysis. Before sample injection, the column was equilibrated with a mixture of 60% Eluent A (300 mM sodium hydroxide solution) and 40% Eluent B (200 mM sodium hydroxide and 170 mM sodium acetate solution). Separation was performed with a flow rate of 1 mL min^−1^ for 25 min using Eluent C, which was ultra-pure water. The quantification of the monosaccharides was performed using an external calibration standard ranging from 0.5 to 30 mg L^−1^. Each sample was analyzed in triplicate. Evaluation of HPAEC-PAD data was carried out using the Chromeleon 7.1 software (Thermo Fisher Scientific).

#### Analysis of ethanol, glycerol and acetate using HPLC-RID

For most series, ethanol (and in some cases glycerol and acetate) was quantified using an Ultimate 3000 HPLC system (Thermo Fisher Scientific) with RID. The separation was carried out with an Aminex HPX-87H column (Bio-Rad Laboratories AB, Solna, Sweden). The mobile phase was an aqueous solution of 0.005 M H_2_SO_4_, with a 0.6 mL min^−1^ flow rate. Both the column oven and the detector were maintained at a temperature of 55 °C. An external calibration curve ranging from 0.05 g L^−1^ to 25 g L^−1^ was employed for quantification. Data analysis was conducted by using the software Chromeleon 7.1.

For Series II (IIa and IIb) and IIIa, ethanol (and in some cases glycerol and acetate) was determined by using a 1260 Infinity HPLC system (Agilent, Santa Clara, CA) with an Aminex HPX-87H column (300 mm × 7.8 mm) equipped with a 125–0131 Standard Cartridge Holder guard column (30 mm × 4.6) (Bio-Rad). The temperature of the column oven and the detector was set to 55 ºC. The injection volume was 10 μL. The eluent, Eluent D, consisted of 0.005 M H_2_SO_4_ and the flow rate was 0.6 mL min^−1^. External calibration curves in the range 0.5 g L^−1^ to 25 g L^−1^ were used for quantification. The software Chromeleon 7.3 was used for the data evaluation.

#### Analysis of aldehydes and ketones using LC–MS/MS

Ultra-high-performance liquid chromatography-electrospray ionization-triple quadrupole-mass spectrometry (UHPLC-ESI-QqQ-MS) was used for the quantification of formaldehyde, acetaldehyde, 4-hydroxybenzaldehyde, coniferyl aldehyde, vanillin, benzoquinone, acetovanillone, furfural, and HMF. Derivatization with 2,4-dinitrophenylhydrazine (DNPH) was performed as previously described [29, 32]. The analysis was performed on an Agilent 1290 Infinity system coupled with an Agilent 6490 Triple Quad mass spectrometer, utilizing electrospray ionization (ESI) in negative mode. The operational parameters were set as follows: gas temperature at 290 °C, gas flow rate at 20 L min^−1^, sheath gas temperature at 400 °C, and sheath gas flow rate at 12 L min^−1^. Separation was achieved using a Kinetex biphenyl column (2.1 mm × 50 mm, 1.7 µm, 100 Å, Phenomenex, Torrance, CA) maintained at 30 °C, with a flow rate of 0.3 mL min^−1^. The mobile phase consisted of two eluents: Eluent E (0.1% formic acid in water) and Eluent F (a mixture of acetonitrile and 2-propanol in a 3:1 ratio, containing 0.1% formic acid). Data analysis was performed using MassHunter Quantitative Analysis software (Agilent).

#### Analysis of methanol using GC-FID

Quantification of methanol was carried out using an Agilent 6890N gas chromatograph (GC) system equipped with a flame ionization detector (FID). The chromatographic separation was achieved using an Agilent HP-INNOWax polyethylene glycol capillary column (30 m × 250 μm i.d., film thickness 0.25 μm). A sample volume of 2 μL was injected in splitless mode using an Agilent 7683 Series automatic liquid sampler. The oven temperature program was set as follows: the initial temperature, 50 °C, was held for 4 min, which was followed by a temperature ramp of 10 °C/min up to180 °C, a further increase up to 250 °C using the same temperature ramp, and then maintained at 250 °C for 5 min. The detector temperature was set to 250 °C. Helium was used as the carrier gas using a constant flow rate of 1.3 mL/min. The flame ionization detector was operated with an air flow rate of 450 mL/min and a hydrogen flow rate of 40 mL/min.

Methanol calibration standards were prepared in a 1:9 (v/v) mixture of ultrapure water and acetone, covering the concentration range from 5 to 1000 ppm. Each calibration standard consisted of 1 mL of the methanol standard solution and 50 µL of an internal standard solution. The internal standard solution was prepared by diluting 3 mL of acetonitrile to a final volume of 100 mL using ultrapure water. For sample preparation, 100 µL of the sample was mixed with 900 µL of acetone to achieve a final volume of 1 mL in a vial. Subsequently, 50 µL of the internal standard solution was added to each sample. The sample mixtures were filtered through a 0.20 µm nylon membrane filter (Merck Millipore Ltd.) prior to analysis. Each sample was analyzed in triplicate. Data evaluation was performed using the ChemStation software (Agilent).

The methanol determination method mentioned above was compared to methanol analysis results achieved by a commercial analytical laboratory (RISE Research Institutes in Sweden AB, Department of Sustainable Materials and Packaging, Unit Pulping Processes, Örnsköldsvik, Sweden), which achieved similar values.

#### Determination of total phenolic content (TPC)

Folin-Ciocalteu’s reagent [34] was used to determine the total phenolic content (TPC) in the laccase experiment in Series IV. Vanillin was used as the calibration standard, the incubation time was 40 min (at room temperature, ~ 23 °C). The reaction mixture was then analyzed by using a BioTek Epoch Microplate Spectrophotometer (Agilent) at λ 760 nm. Measurements were conducted in triplicate.

## Data Availability

The datasets used and/or analyzed during the current study are available from the corresponding author on reasonable request.
